# Supplementary material for: Direct binding of TFEα opens DNA binding cleft of RNA polymerase
Source: Nat Commun. 2020 Nov 30;11:6123. doi: 10.1038/s41467-020-19998-x (PMC7704642; doi:10.1038/s41467-020-19998-x)
Supplement: Supplementary file 1 — Supplementary information [file 41467_2020_19998_MOESM1_ESM.pdf]

Supplementary information for

Direct binding of TFEα opens DNA binding cleft of RNA polymerase

Jun et al.,

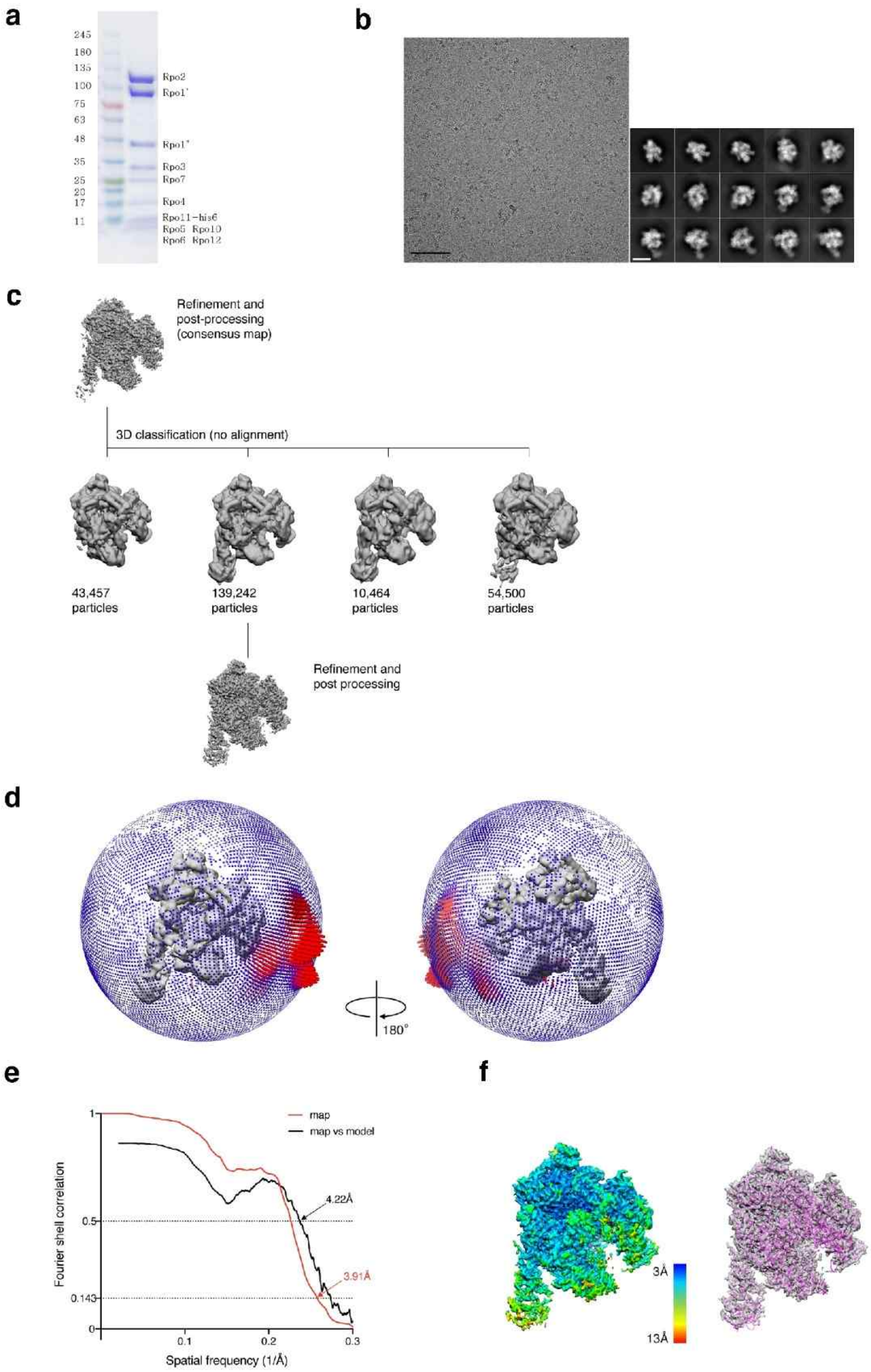
a
245
180
135
100
75
63
48
35
25
20
17
11
Rpo2
Rpo1'
Rpo1"
Rpo3
Rpo7
Rpo4
Rpo11-his6
Rpo5 Rpo10
Rpo6 Rpo12
b
c
Refinement and post-processing (consensus map)
3D classification (no alignment)
43,457 particles
139,242 particles
10,464 particles
54,500 particles
Refinement and post processing
d
180°
e
map
map vs model
Fourier shell correlation
1
0.5
0.143
0
4.22Å
3.91Å
0.1
0.2
0.3
Spatial frequency (1/Å)
f
3Å
13Å

Supplementary Fig. 1. Cryo-EM analysis of apo-RNAP

a, SDS-PAGE analysis of apo-RNAP. Left lane: molecular weight marker (kDa). The results were reproducible in more than five independent experiments.

b, Representative cryo-electron micrograph (left) and 2D class averages (right). Scale bars are 50 nm and 5 nm in the micrograph and class averages, respectively. We could see similar results in more than three independent experiments.

c, 3D classification and refinement procedures.

d, Angular orientation distribution of the final 3D reconstruction.

e, FSC between independently refined half maps and cross FSC between cryo-EM map and model shown in red and black curves, respectively

f, Local resolution map (left) and C_{α} trace of the atomic model (magenta) docked into the EM map (right).

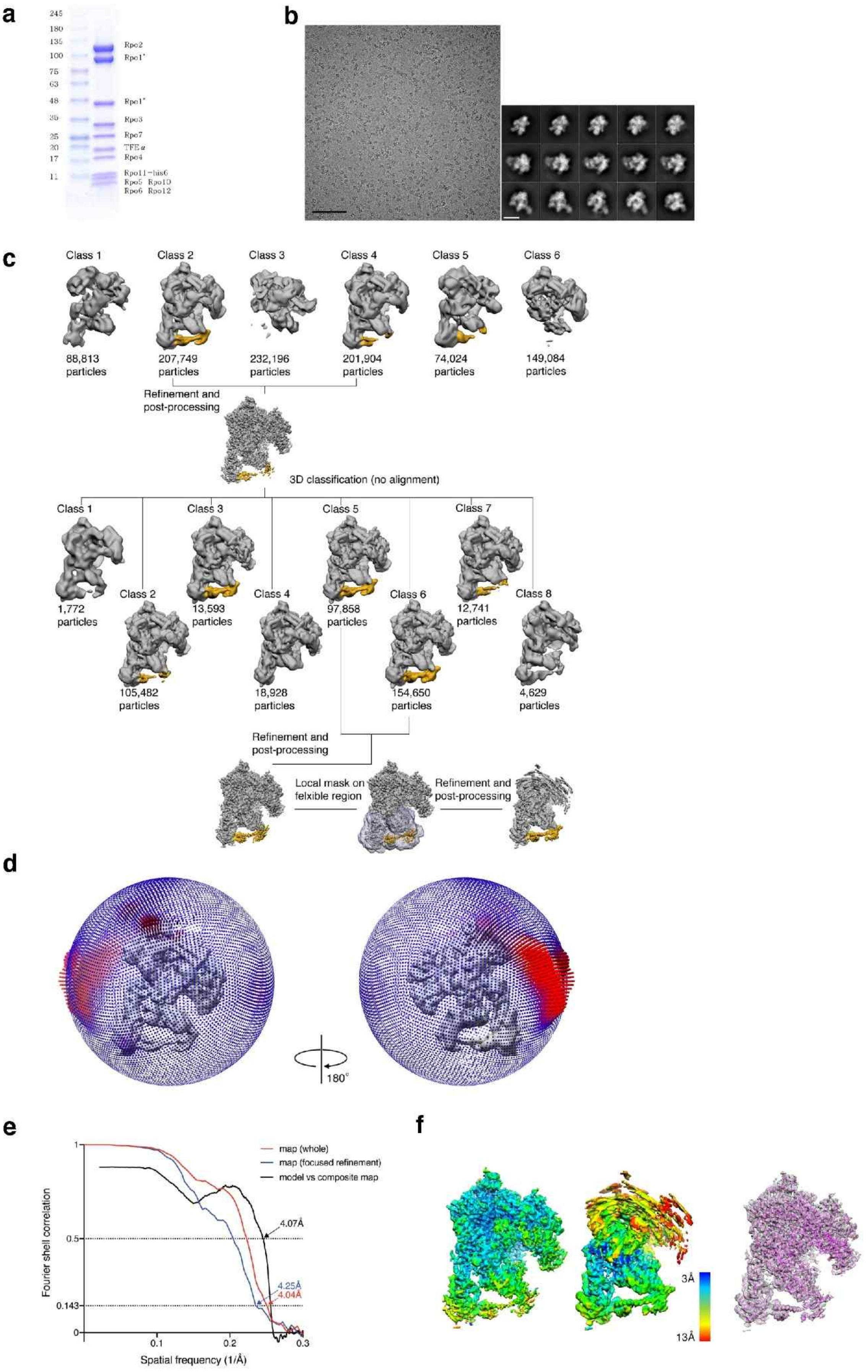
a
245
180
135
100
75
63
48
35
25
20
17
11
Rpo2
Rpo1'
Rpo1''
Rpo3
Rpo7
TFEα
Rpo4
Rpo11-his6
Rpo5 Rpo10
Rpo6 Rpo12
b
c
Class 1
Class 2
Class 3
Class 4
Class 5
Class 6
88,813 particles
207,749 particles
232,196 particles
201,904 particles
74,024 particles
149,084 particles
Refinement and post-processing
3D classification (no alignment)
Class 1
Class 2
Class 3
Class 4
Class 5
Class 6
Class 7
Class 8
1,772 particles
105,482 particles
13,593 particles
18,928 particles
97,858 particles
154,650 particles
12,741 particles
4,629 particles
Refinement and post-processing
Local mask on felxible region
Refinement and post-processing
d
180°
e
map (whole)
map (focused refinement)
model vs composite map
4.07Å
4.25Å
4.04Å
Fourier shell correlation
1
0.5
0.143
0
0.1
0.2
0.3
Spatial frequency (1/Å)
f
3Å
13Å

Supplementary Fig. 2. Cryo-EM analysis of RNAP-TFEα

a, SDS-PAGE analysis of RNAP-TFEα binary complex. Left lane: molecular weight marker (kDa). The results were reproducible in more than five independent experiments.

b, Representative cryo-electron micrograph (left) and 2D class averages (right). Scale bars are 50 nm and 5 nm in the micrograph and class averages, respectively. We could see similar results in more than three independent experiments.

c, 3D classification strategy to select particle images of intact RNAP-TFEα binary complex and procedures employed to perform 3D refinement of the whole complex and focused refinement on region containing stalk and clamp domains of RNAP and TFEα. EM map density that corresponds to TFEα is shown in yellow.

d, Angular orientation distribution of the final 3D reconstruction.

e, FSC between independently refined half maps of the whole complex, half maps from focused refinement, and cross-FSC between cryo-EM map and the refined model are shown in red, blue and black curves, respectively (left),

f, Local resolution maps of the whole complex (left) and focused map (middle), and C_α trace of the atomic model (magenta) docked into the composite EM map (right).

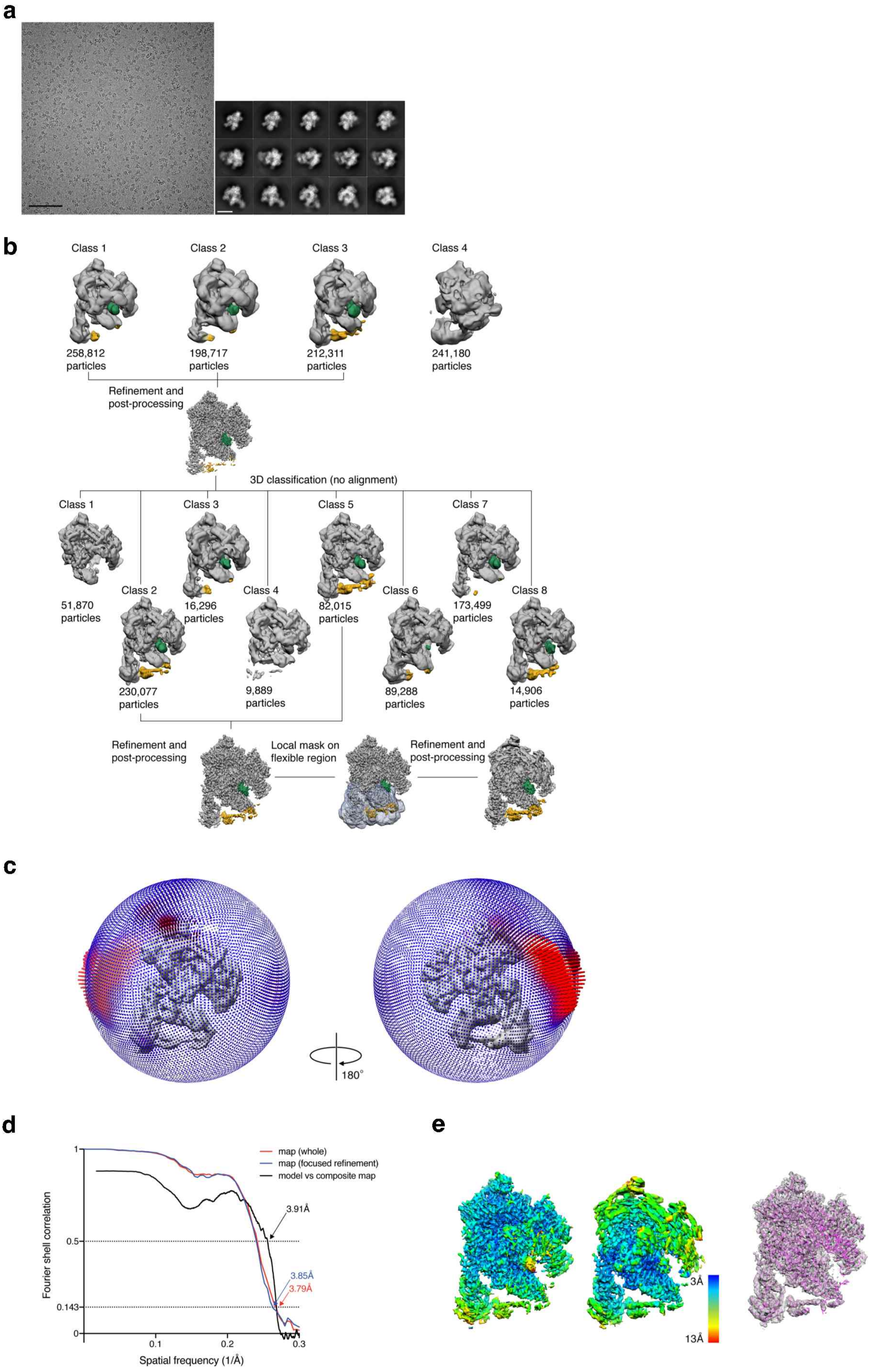
a
b
Class 1
Class 2
Class 3
Class 4
258,812 particles
198,717 particles
212,311 particles
241,180 particles
Refinement and post-processing
3D classification (no alignment)
Class 1
Class 3
Class 5
Class 7
Class 2
Class 4
Class 6
Class 8
51,870 particles
16,296 particles
82,015 particles
173,499 particles
230,077 particles
9,889 particles
89,288 particles
14,906 particles
Refinement and post-processing
Local mask on flexible region
Refinement and post-processing
c
180°
d
map (whole)
map (focused refinement)
model vs composite map
3.91Å
3.85Å
3.79Å
Fourier shell correlation
Spatial frequency (1/Å)
1
0.5
0.143
0
0.1
0.2
0.3
e
3Å
13Å

Supplementary Fig. 3. Cryo-EM analysis of RNAP-TFEα-DNA

a, Representative cryo-electron micrograph (left) and 2D class averages (right). Scale bars are 50nm and 5nm in the micrograph and class averages, respectively. We could see similar results in more than three independent experiments.

b, 3D classification strategy to select particle images of intact RNAP-TFEα-DNA ternary complex and procedures employed to perform 3D refinement of the whole complex and focused refinement on region containing stalk and clamp domains of RNAP and TFEα. EM map density that corresponds to TFE and DNA are shown in yellow and green, respectively.

c, Angular orientation distribution of the final 3D reconstruction.

d, FSC between independently refined half maps of the whole complex, half maps from focused refinement, and cross-FSC between cryo-EM map and the refined model are shown in red, blue, and black curves, respectively (left),

e, Local resolution maps of the whole complex (left) and focused map (middle), and C_α trace of the atomic model (magenta) docked into the composite EM map (right).

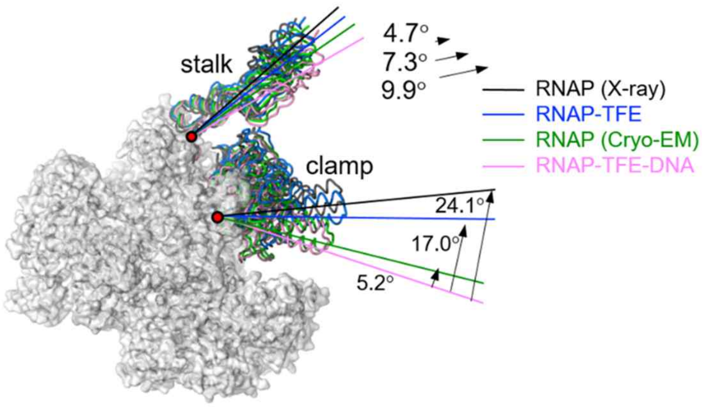

Supplementary Fig. 4. RNAP clamp and stalk conformations.

The RNAP is shown as surface (white) and four different states of the clamp and stalk are shown as ribbon. Angles of clamp and stalk swinging are shown.

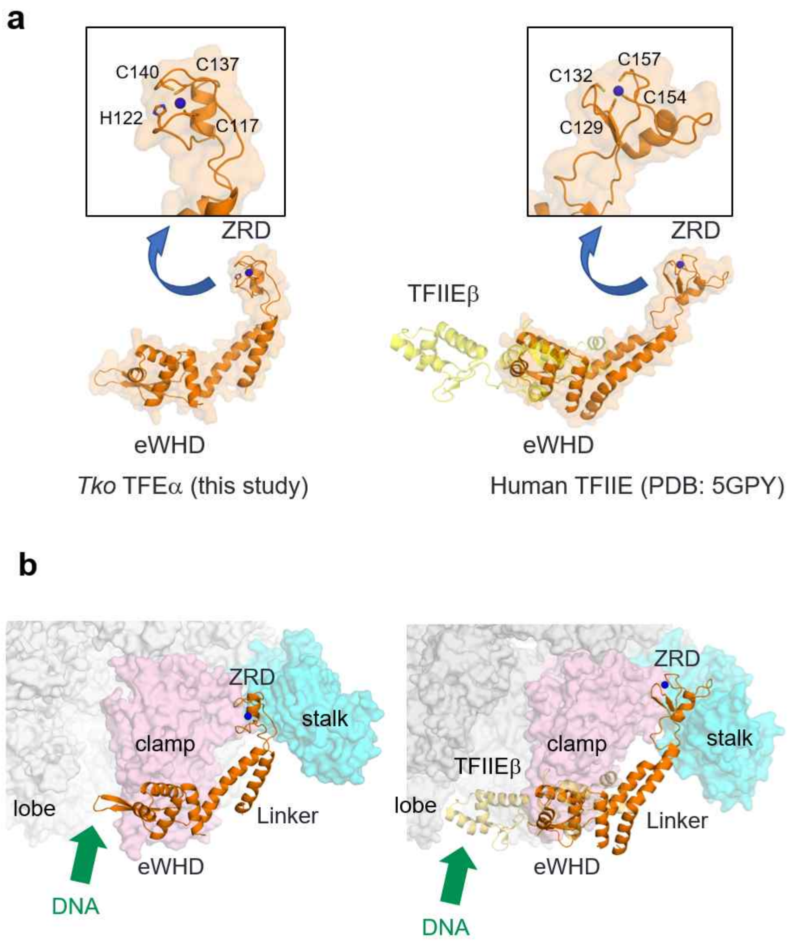

Supplementary Fig. 5. Comparison of the structures of archaeal TFEα and eukaryotic TFIIEα.

a, Structures of the *Tko* TFEα (left) and the yeast TFIIEα (right) are shown as ribbon models with transparent surfaces. In the yeast TFIIE, the structure of TFIIEβ is also shown as a transparent ribbon model. Magnified views of the ZBDs of *Tko* TFEα and human TFIIEα are shown in boxes. Amino acid residues coordinating Zn atom (blue sphere) are shown and labeled.

b, Comparison between the archaeal *Tko* RNAP-TFEα complex (left) and the eukaryotic human Pol II and TFIIE complex (right). DNA binding main clefts of RNAPs are indicated by green arrows.

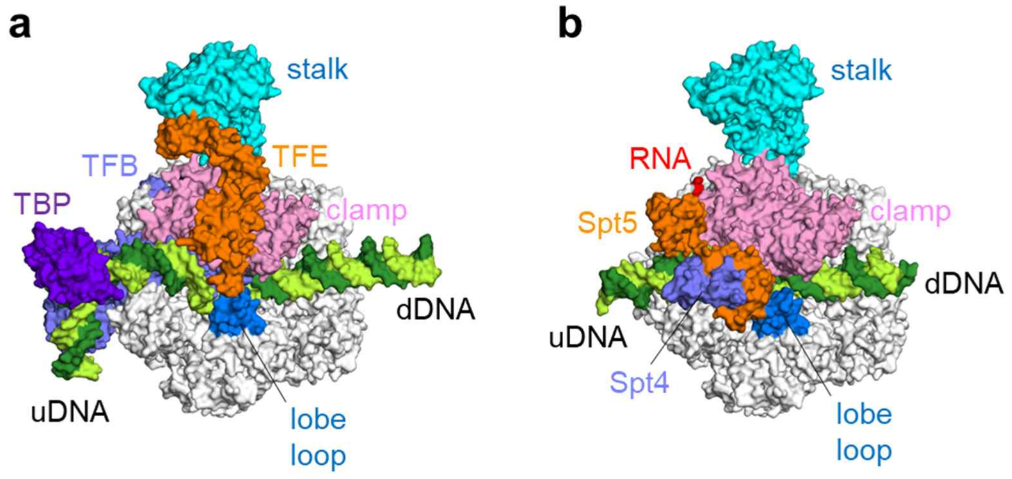

Supplementary Fig. 6. Comparison of the TFEα and Spt4/5 interactions with RNAP

a, The OC model as shown in Fig. 5b.

b, The archaeal transcription elongation complex model with Spt4/5. The Spt4/5 is modeled using the cryo-EM structure of *Pfu* RNAP and Spt4/5 complex (EMD ID: 1840), the crystal structure of *Pfu* Spt4/5 (PDB: 3P8B) and the cryo-EM structure of the Pol II EC with Spt4/5 (PDB: 5XON) and as references. Proteins and domains are indicated.

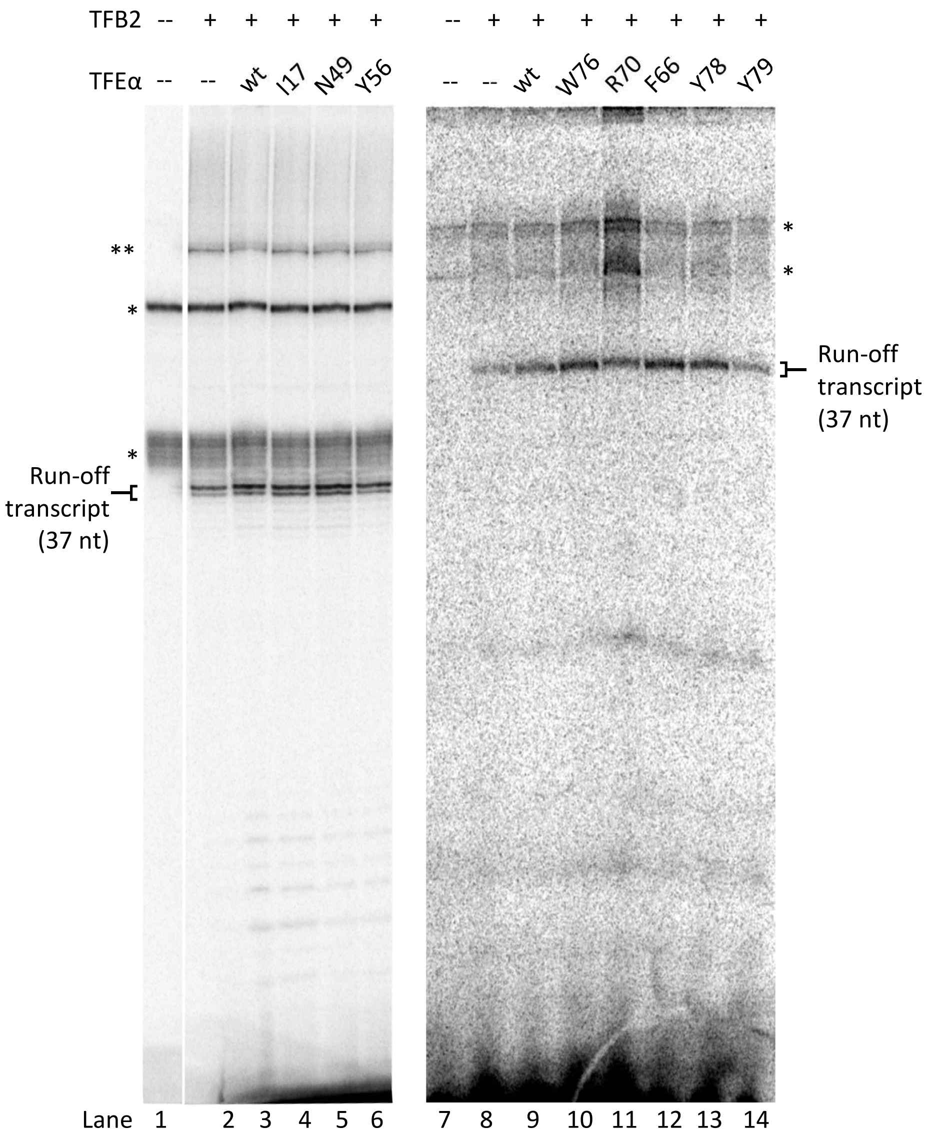

Supplementary Fig. 7. Transcription activation by TFEα variants containing Bpa substitutions. The *Pfu gdh* promoter (-60 to +37) was transcribed with RNAP, TBP, TFB2, and wild type TFEα or TFEα variants containing Bpa at the positions indicated. The position of the run-off transcript (37 nt) is indicated. The run-off doublet observed in lanes 1-6 likely arises from heterogeneity in the downstream end of the PCR product used as a promoter template. A different lot of primers was used to generate the promoter template used in lanes 7-14 and yielded a singlet transcript. Recovery marker DNA bands (97 nt) are indicated by the asterisk. The higher molecular weight band observed in lanes 2-6 (indicated by the double asterisk) is likely from end-to-end template switching.

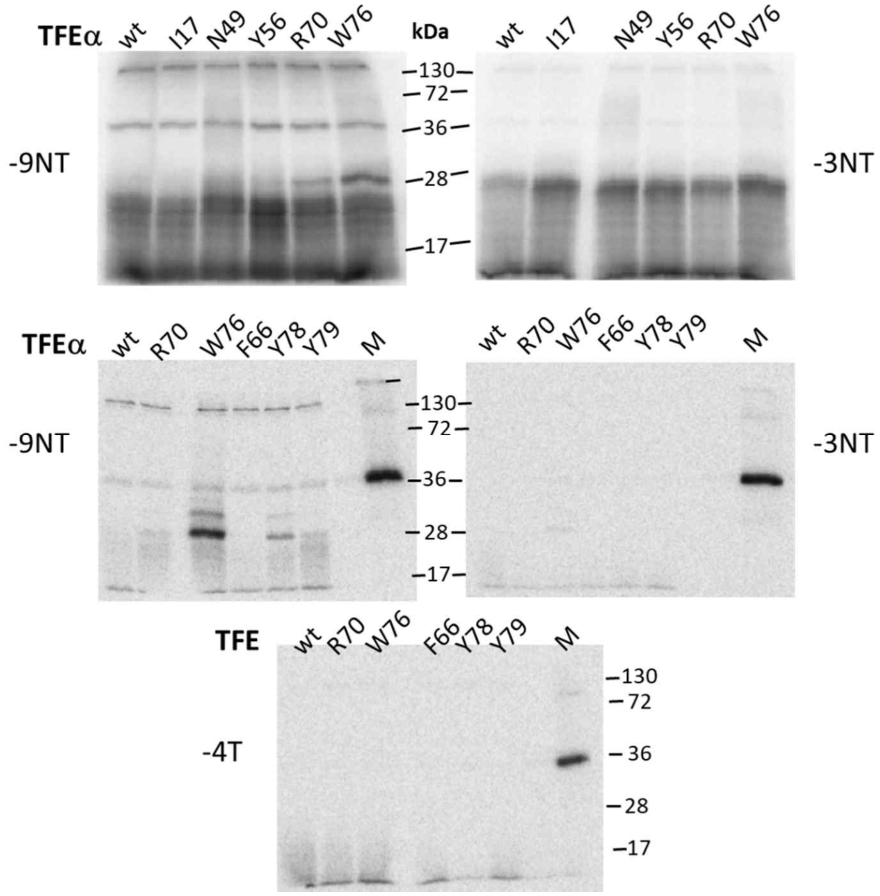

Supplementary Fig. 8. Cross-linking of Bpa-containing TFEα variants to selected residues in the T and NT strands. Proteins in transcription initiation complexes were separated by SDS-PAGE following cross-linking and nuclease treatment. TFEα variants are listed at the top of each gel, and the radiolabel positions at the sides. TFEα runs at about 28 kDa in these gels. In -9NT gels, background labeling of higher molecular weight proteins (likely an RNAP subunit, and TFB) was observed. In the second -9NT gel, weak bands just above TFEα (at 28 kDa) in W76 and Y78 likely arise from a small fraction of TFEα attached to undigested DNA. In the top two gels, the smear at~20 kDa and smaller is likely undigested probe DNA. In the bottom three gels, the M lane (Marker) shows TFB1 F192-Bpa cross-links to DNA labeled at -19NT, which was previously observed to give strong cross-links at ~38 kDa. The results were reproducible in more than three independent experiments.

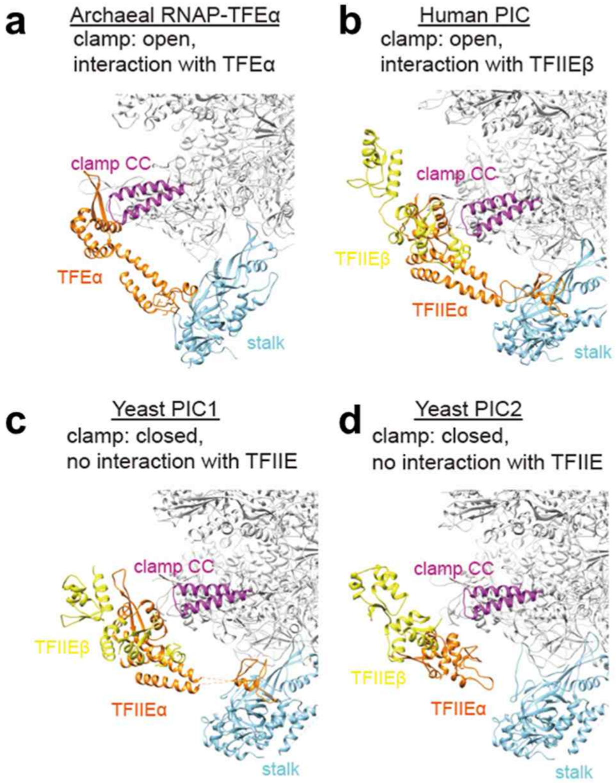

Supplementary Fig. 9. Comparison of archaeal RNAP-TFEα complex and Pol II PIC structures. Clamp conformation and its interaction with TFEα/TFIIE are denoted.

a, *Tko* RNAP-TFEα complex at 4.0 Å.

b, Human PIC at 5.4 Å (PDB: 5IYA)[16].

c, Yeast PIC at 8.8 Å (PDB: 5FZ5)[15].

d, Yeast PIC at 6.0 Å (PDB: 5FMF)[17].

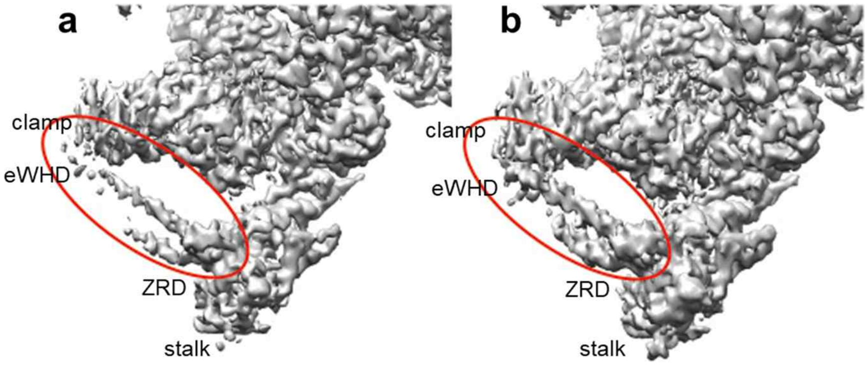

Supplementary Fig. 10. Cryo-EM of TFEα in RNAP-TFEα binary complex in high (a) and low (b) thresholds. Density of TFEα is indicated by a red ellipse. Domains of TFEα and RNAP are denoted.

Supplementary Table 1. X-ray crystallographic data collection and refinement statistics.

	TFEα SeMet	TFEα native
PDB code	6XJF	6PLN
**Data collection**		
Wavelength	0.977	0.977
Space group	$P2_12_12_1$	$P4_12_12$
a, b, c (Å)	76.199, 115.61, 124.209	55.689, 55.689, 248.673
α, β, γ (°)	90, 90, 90	90, 90, 90
Resolution (Å)	44.4 – 3.2<br>(3.3 – 3.2)*	4.0 – 2.6<br>(2.64 – 2.6)*
R_{merge} (%)	7.4 (93.5) *	6.4 (99.1)*
I / σI	19.2 (3.1) *	59.1 (3.6)*
Completeness (%)	99.99 (99.89)*	99.9 (100.0)*
Redundancy	4.4 (2.7)*	13.1 (12.8)*
**Refinement**		
Resolution (Å)	44.4 – 3.2	33.3 – 2.6
No. reflections	18,695	170,860
R_{work} / R_{free} (%)	24.7/30.6	21.3/24.6
**No. atoms**		
Protein	6,810	1,724
Water	0	18
***B* factors (Å^2)**		

Protein	82.4	85.5
Water		56.9
**r.m.s. deviations**		
Bond lengths (Å)	0.013	0.003
Bond angles (°)	1.87	0.56
**Ramachandran plot**		
Favored (%)	98.39	99.02
Allowed (%)	1.61	0.98
Outliers (%)	0.0	0.0

*Highest resolution shell is shown in parenthesis

Supplementary Table 2. Cryo-EM data collection and refinement statistics.

	Apo-form (EMD-9960) (PDB 6KF3)	RNAP-TFEα (EMD-9961) (PDB 6KF4)	RNAP-TFEα-DNA (EMD-9962) (PDB 6KF9)
**Data collection and processing**			
Magnification	100,000	100,000	100,000
Voltage (kV)	300	300	300
Electron exposure (e–/Å^2)	35	35	35
Defocus range (µm)	-1.5 to -3.5	-1.5 to -3.5	-1.5 to -3.5
Pixel size (Å)	1.4	1.4	1.4
Symmetry imposed	C1	C1	C1
Initial particle images (no.)	514,007	1,226,339	1,388,666
Final particle images (no.)	139,242	409,653	312,092
Map resolution (Å)*	3.91	4.04	3.79
FSC threshold	0.143	0.143	0.143
Map resolution range (Å)	3.33-12.97	3.33-12.32	3.33-12.97
**Refinement**			
Initial model used (PDB code)	4QIW	4QIW	4QIW
Model resolution (Å)**	4.22	4.07	3.91
FSC threshold	0.5	0.5	0.5
Map sharpening *B* factor (Å^2)	-140.08	-185.58	-144.71
Model composition			
Non-hydrogen atoms	26,374	27,746	28,231
Protein residues	3,297	3,462	3,463
Nucleic acid residues	0	0	23
Ligands	6	7	7
B factors (Å^2)			
Protein	38.75	61.82	43.61
Nucleotide	-	-	113.92
Ligand	82.00	108.70	78.50
R.m.s. deviations			
Bond lengths (Å)	0.008	0.010	0.007
Bond angles (°)	1.332	1.573	1.252
Validation			
MolProbity score	2.39	2.46	2.39
Clashscore	4.48	5.19	4.54
Poor rotamers (%)	4.74	4.54	4.60
Ramachandran plot			
Favored (%)	85.25	83.54	85.20
Allowed (%)	13.92	13.55	13.81
Disallowed (%)	0.82	2.91	0.99

*Resolution of whole map refinement is shown. Resolution of the map resulting from focused refinement is shown in Supplementary Figs. 2c and 3c.

**Model resolution is based on composite EM map from whole map refinement and focused refinement (Supplementary Figs. 2d and 3d)

Supplementary Table 3. Polymerase Chain Reaction (PCR), mutagenesis, and cross-linking probe primers and oligos

	Primer/oligo	sequence
Pfu TFEαΔZRD	Forward primer	5'-GGTCTCAAGGTATGGGCAGAGACAAAAAAAATAC-3'
	Reverse primer	5'-AATTCTCGAGTTATTCTTCCTGAAGCATCTGCTT-3'
Pfu TFEαΔZRD mutagenesis	I13 sense	5'-AGCGCTTCTAGATATGGCAAGAGATATAG-3'
	I13 antisense	5'-CTATATCTCTTGCCATATCTAGAAGCGCT-3'
	I54 sense	5'-TACGGTGAGAAAAATGTTATACGCCCTGT-3'
	I54 antisense	5'-ACAGGGCGTATAACATTTTTCTCACCGTA -3'
	F66 sense	5'-TAAGCTTGCAACCATGAGAAGAGTTAGAG-3'
	F66 antisense	5'-CTCTAACTCTTCTCATGGTTGCAAGCTTA-3'
Tko TFEα	Forward primer	5'-AATTCGTCTCAAGGTATGGCAAAGCGCAAGAACAAG-3'
	Reverse primer	5'-CGCGGATCCTTATCACTCAGACCTTGACTTCTTTTT-3'
Tko gdh promoter	Template strand	5'-TCGGTAATCACGCTCC-3'
	Non-Template strand	5'-GCCTAAGTTAACCTCGCAGATTACCGA-3'
Pfu gdh promoter, -60 to +37	Forward primer	5'-AAAGGATTTCCACTCTTGTTTACC-3'
	Reverse primer	5'-CTCAACCATGTTCATCCCTC-3'
	Forward primer biotinylated	5'-BIO-CATGCATGTTTAAAGGATTTCCACTCTTGTTTAC-3'
	Reverse primer biotinylated	5'-BIO-CATGCATGCCGCTCAACCATGTTCATCCCTCC-3'
Pfu TFE mutagenesis	I17 sense	5'-GCGCTTCTAGATATTGCAAGAGATTAGGGAGGAGATGAAGCTGTAGAAG-3'
	I17 antisense	5'-CTTCTACAGCTTCATCTCCTCCCTAATCTCTTGCAATATCTAGAAGCGC-3'
	N49 sense	5'-GAATTGGCAGAATTAACTGGAGTAAGAGTTTAGACGGTGAGAAAAATCTTA-3'
	N49 antisense	5'-TAAGATTTTTCTCACCGTCTAAACTCTTACTCCAGTTAATTCTGCCAATTC-3'
	Y56 sense	5'-AGTTAATACGGTGAGAAAAATCTTATAGGCCCTGTACGATGC-3'
	Y56 antisense	5'-GCATCGTACAGGGCCTATAAGATTTTTCTCACCGTATTAACT-3'
	F66 sense	5'-TACGATGCTAAGCTTGCAACCTAGAGAAGAGTTAGAGATGACG-3'
	F66 antisense	5'-CGTCATCTCTAACTCTTCTCTAGGTTGCAAGCTTAGCATCGTA-3'
	R70 sense	5'-CTAAGCTTGCAACCTTTAGAAGAGTTTAGGATGACGAGACTGGTTGGTATTATTA-3'
	R70 antisense	5'-TAATAATACCAACCAGTCTCGTCATCCTAAACTCTTCTAAAGGTTGCAAGCTTAG-3'
	W76 sense	5'-AGAGATGACGAGACTGGTTAGTATTATTATTACTGGCGC-3'
	W76 antisense	5'-GCGCCAGTAATAATAATACTAACCAGTCTCGTCATCTCT-3'
	Y78 sense	5'-TGACGAGACTGGTTGGTATTAGTATTACTGGCGCATTG-3'
	Y78 antisense	5'-CAATGCGCCAGTAATACTAATACCAACCAGTCTCGTCA-3'
	Y79 sense	5'-TGACGAGACTGGTTGGTATTATTAGTACTGGCGCATTG-3'
	Y79 antisense	5'-CAATGCGCCAGTACTAATAATACCAACCAGTCTCGTCA-3'
Pfu gdh promoter cross-link probes	-9NT	5'-AAAGGATTTCCACTCTTGTTTACCGAAAGCTTTATATAGGCTATTGCCCAA-3'
	-3NT	5'-AAAGGATTTCCACTCTTGTTTACCGAAAGCTTTATATAGGCTATTGCCCAAAAATGT-3'
	-4T	5'-CTCAACCATGTTCATCCCTCCAAATTAGGTGATTGGCGAT-3'
