# Supplementary material for: Direct binding of TFEα opens DNA binding cleft of RNA polymerase
Source: Nat Commun. 2020 Nov 30;11:6123. doi: 10.1038/s41467-020-19998-x (PMC7704642; doi:10.1038/s41467-020-19998-x)
Supplement: Supplementary file 3 — Description of Additional Supplementary Files [file 41467_2020_19998_MOESM3_ESM.pdf]

Description of Additional Supplementary Files

Supplementary Movie 1: Conformational changes of *Tko* RNAP

This video shows conformational changes in *Tko* RNAP that occur during formation of the binary complex (from the apo-form RNAP to RNAP-TFEα complex) and the ternary complex (from the binary to RNAP-TFEα-DNA complex). The movie is related to Fig. 2.

Supplementary Movie 2: PIC and open complex formations

This video shows the PIC and open complex formations in the archaeal transcription system in the presence of TFEα.
